# Effect of output intensity of light curing unit on coronal tooth discoloration induced by silicate calcium-based materials: an ex vivo study

**DOI:** 10.2340/biid.v13.45896

**Published:** 2026-05-08

**Authors:** Fatemeh Farshad, Noushin Shokouhinejad, Parham Pedram, Mohammad Javad KharaziFard, Elham Ahmadi

**Affiliations:** a Student’s Scientific Research Center, Tehran University of Medical Education, Tehran, Iran; b Department of Endodontics, School of Dentistry, Tehran University of Medical Sciences, Tehran, Iran; c Department of Dental Biomaterial, School of Dentistry, Shahid Beheshti University of Medical Sciences, Tehran, Iran; d Dental Research Center, Dentistry Research Institute, Tehran University of Medical Sciences, Tehran, Iran

**Keywords:** Dental curing light, mineral trioxide aggregate, tooth discoloration

## Abstract

**Objective:**

Clinicians have various curing options due to new light-cure devices with different intensities. The present study aimed to evaluate the effect of two light-curing intensities (1,200 mW/cm² vs. 3,000 mW/cm²) on coronal discoloration induced by two calcium silicate-based cements (OrthoMTA and RetroMTA) in an endodontically treated bovine tooth model.

**Materials and Methods:**

Forty single-rooted bovine incisors were prepared, and their canals were filled with blood-soaked foam to the cemento-enamel junction. The samples were randomly divided into four groups (*n* = 10 per group) based on the cement used (OrthoMTA or RetroMTA) and the subsequent light-curing intensity. For all groups, a 2-mm layer of the assigned cement was placed. This was covered by 1 mm of resin-modified glass ionomer (RMGI), a bonding agent, and 2 mm of composite. In two groups, all light-curing steps (RMGI, bonding agent, composite) were performed at 1,200 mW/cm². In the other two groups, curing was performed at 3,000 mW/cm². All samples underwent 5,000 cycles of thermocycling. Color was assessed with a spectrophotometer immediately after composite placement and after aging. Color change (ΔE) and lightness change (ΔL) were calculated.

**Results:**

All groups exhibited ΔE values exceeding the clinically acceptable threshold (ΔE > 3.3) and demonstrated a decrease in ΔL after aging. Statistical analysis (two-way ANOVA, α = 0.05) revealed no significant difference in ΔE or ΔL between samples cured with high-intensity (3,000 mW/cm²) or low-intensity (1,200 mW/cm²) light, regardless of the cement type used (*p* > 0.05).

**Conclusions:**

Within the limitations of this study, the same amount of energy produced by high- or low-intensity light-cures (1,200 or 3,000 mW/cm²), when delivering the same total energy, did not result in a statistically significant difference in coronal discoloration caused by OrthoMTA or RetroMTA.

## Introduction

The first hydraulic calcium silicate-based cement (HCSC) was ProRoot mineral trioxide aggregate (MTA) (Dentsply, Tulsa Dental Specialties, Tulsa, OK, USA), introduced in endodontics by Torabinejad in 1993 as a root-end filling material [1]. Since then, its applications have extended into multiple aspects, including repair of root perforations, apexification, apexogenesis, vital pulp therapy (VPT), and regenerative endodontic treatment, due to its favorable properties [2, 3]. Its use as a coronal barrier in pulp capping and pulpotomy procedures is standard, as it provides a bioactive seal over the pulp and a stable, dentin-like interface, which is crucial for long-term success and the prevention of microleakage [4].

Although ProRootMTA has notable benefits, such as excellent biocompatibility and sealing ability, it also has several limitations. These limitations include its challenging handling, high cost, and tooth discoloration over time [2, 5]. Several studies illustrated potential reasons for discoloration. The interaction of MTA with blood during its hydration process might play a role in causing discoloration [6]. The penetration of blood components into the porosities of HCSC materials could be a possible mechanism [3]. In addition, materials containing bismuth oxide as a radiopacifier had high staining potential [7]. It has been shown that the destabilization of bismuth oxide due to exposure to light irradiation [8] and strong oxidizing agents led to the discoloration of HCSCs [9]. The color change is presumably due to the conversion of yellow bismuth oxide to dark metallic bismuth or bismuth carbonate [8, 9]. Moreover, it has been shown that regardless of the presence of added carbon dioxide, discoloration happens when exposed to light, with the intensity increasing over time [10]. The change in the color of bismuth oxide-containing materials and their adjacent tooth structure is more likely than that of bismuth oxide-free substances [3]. Therefore, attempts to find materials with less potential for tooth discoloration and better color stability have led to the introduction of HCSCs with alternative radiopacifiers such as zirconium oxide, tantalum oxide, or barium sulphate [3].

Given the effect of light on HCSCs, dental equipment can also contribute to tooth discoloration. Dentists’ routine use of light-emitting diode (LED) light-cure devices to cure filling materials, including bonding agents and composites, might also impact the color properties of HCSC. High-power LEDs are a new generation of light-cure devices that decrease clinical time management by reducing exposure time [11]. New high-energy light sources may reduce radiation time. However, the effect of such a change on temperature conduction to the pulp remains unclear [12].

Clinicians have various curing options due to new technologies and light-cure devices with different intensities. In addition, considering the favorable clinical results of HCSC in pulp therapy treatments, these materials are increasingly used in dentistry. Therefore, the present study aimed to evaluate the color change of teeth treated with a bismuth oxide-containing material (OrthoMTA) and a bismuth oxide-free material (RetroMTA) with two intensities of LED Light-cure. To the best of our knowledge, this is the first study that evaluates the effect of LED light-cure intensity on tooth color changes.

## Materials and methods

The manuscript of this laboratory study has been written according to the Preferred Reporting Items for Laboratory studies in Endodontology (PRILE) 2021 guidelines.

This ex vivo experimental study adhered to ARRIVE guidelines [13] and adhered to the National Institute of Health’s guide on animal care and use, and received approval from the Ethical Committee of Tehran University of Medical Sciences (No. IR.TUMS.DENTISTRY.REC.1402.046).

A power analysis for a one-way ANOVA was performed using PASS11 software with parameters set to alpha = 0.05, beta = 0.2 (power = 80%), an effect size of 0.57, and a delta (Δ) of 2.2, following the methodology of Camilleri [9]. This analysis determined that a minimum sample size of 10 teeth per group was required.

### Preparation of samples

Forty bovine incisors were selected. The teeth were free of caries or cracks. Debris was removed using manual scaling equipment (Varios 970; NSK, Kanuma-shi, Tochigi, Japan). Then, the teeth were immersed in Chloramine-T 0.5% for 7 days to be disinfected. Subsequently, they were polished with pumice paste and water to remove stains and stored in the artificial saliva at room temperature until needed.

Each tooth was randomly assigned a number from 1 to 40 by the operator. The apical part of each root was removed perpendicular to its long axis with a high-speed diamond fissure bur (#010) (Tizkavan, Tehran, Iran) while continuously spraying water until 5 mm of root remained. Then, access cavities were prepared, and the shortened root canals were cleaned and shaped using #1 to 6 Gates Glidden drills (Dentsply Maillefer, Ballaigues, Switzerland) and rinsed with distilled water.

### Blood collection

Five mL of fresh human blood was collected from a healthy volunteer who consented to participate in the study. An anticoagulant-coated tube was used to prevent clotting, and the blood was used immediately.

### Experimental setup

Customized foam soaked in fresh human blood was inserted into the root canal through the apical opening, extending to the cemento-enamel junction (CEJ). After applying the foams, the root ends were sealed with a resin-modified glass ionomer (RMGI) (Iono Cid-L, Iran).

The 40 teeth were blindly divided into two groups. In each group, either OrthoMTA (BioMTA, Seoul, Korea) or RetroMTA (BioMTA, Seoul, Korea) was mixed with distilled water following the manufacturer’s instructions (Table 1). A layer of the MTA (2 mm) was placed in the coronal area of the root canal on blood-soaked foam above the labial CEJ to simulate the VPT [14]. Then, a wet cotton pellet was placed over the MTA, and the teeth were restored with temporary filling material (Coltosol, Asia Chemi Teb Co., Tehran, Iran). The teeth were incubated at 37°C under fully saturated humidity conditions for 7 days. After this period, the temporary filling material and wet cotton pellet were removed. Finally, each group was randomly divided into two subgroups containing 10 teeth.

**Table 1:** Composition of material and solutions used in brushing simulation.

Name	Composition
OrthoMTA (BioMTA)	Dicalcium silicate, Tricalcium silicate, Tricalcium aluminate, Bismuth oxide (likely), Calcium carbonate.
RetroMTA (BioMTA)	Calcium carbonate, Dicalcium silicate, Tricalcium aluminate, Zirconium oxide, Calcium chloride.
Temporary filling (Coltosol)	Zinc oxide, Zinc sulfate, Polyvinyl acetate, Calcium sulfate, Pigments and flavorants.
RMGI (GC, e.g., Fuji II LC)	Powder: Fluoroaluminosilicate glass.Liquid: Polyacrylic acid, Itaconic acid, Tartaric acid, Water.
Composite Resin (Estelite)	Bis-GMA, TEGDMA, UDMA, Bis-MPEPP, Silanated silica-zirconia filler.

RMGI: resin-modified glass ionomer; Triethylene glycol dimethacrylate (TEGDMA); Urethane dimethacrylate (UDMA).

**Figure 1:**
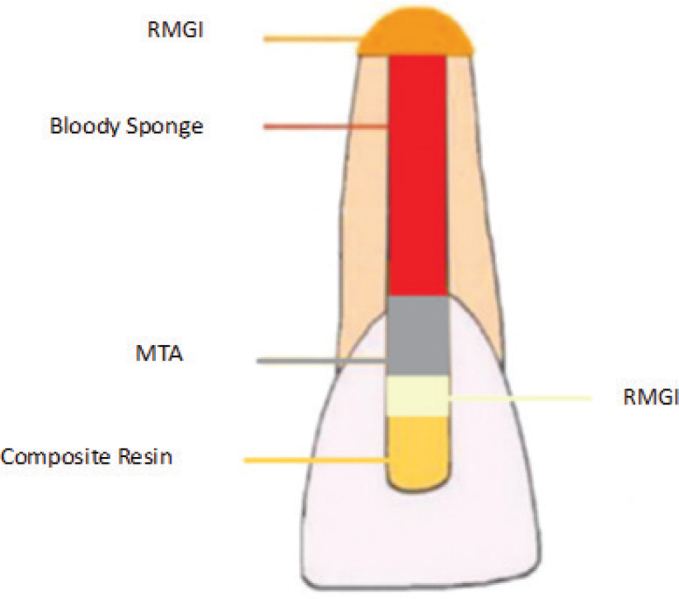
Schematic illustrations of the procedure of this study.

Above the MTA, a 1-mm layer of RMGI (GC, Tokyo, Japan, shade A_2_) was applied. After that, we used a light-curing unit (Woodpecker, China) to set RMGI. Then, a two-bottle self-etch bonding agent (Clearfil SE Bond, Kuraray Noritake Dental Inc., Japan) was applied to the whole access cavity. It was set at the same intensity. Finally, teeth were restored with a composite resin (Estelite, Tokyo, Japan, shade A_2_). We used the same intensity to set the composite resin (Figure 1). The manufacturer’s instructions were followed, which assert that glass ionomer, bonding agent, and composite should receive 20, 10, and 10 s, respectively, with low-intensity light-cure (1,200 mW/cm^2^). As a result, the energy transferred to the teeth was 48,000 J.

**Figure 2:**
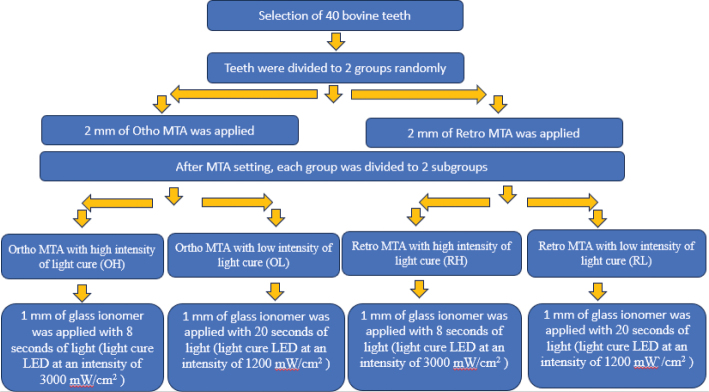
Schematic illustrations of the procedure of each stage in the present study.

To equalize the amount of energy [Work (J) = Power (mW/cm^2^) * time (s)] in all subgroups, the subgroups cured with high-intensity light-cure (3,000 mW/cm^2^) received 8, 4, and 4 s light for a glass ionomer, bonding agent, and composite, respectively (Figure 2).

After light-curing, the composite at the palatal surface of all samples was then finished and polished with a high-speed diamond football-shaped bur super fine (k_1_) (Tizkavan, Tehran, Iran). The samples were rinsed for 10 s between the polishing steps.

### Aging procedure

The samples underwent 5,000 cycles of thermocycling (TC3000, Vafai Industrial Co., Tehran, Iran) between water baths of 5 and 55°C with a 20-s dwell time. The 5,000 cycles of thermocycling are equivalent to 6 months of oral function [15, 16].

### Color assessment

Tooth color measurements were conducted in a dark room using a spectrophotometer (VITA Zahnfabrik, Bad Säckingen, Germany). Before each color measurement, the device was calibrated according to the manufacturer’s instructions.

**Figure 3:**
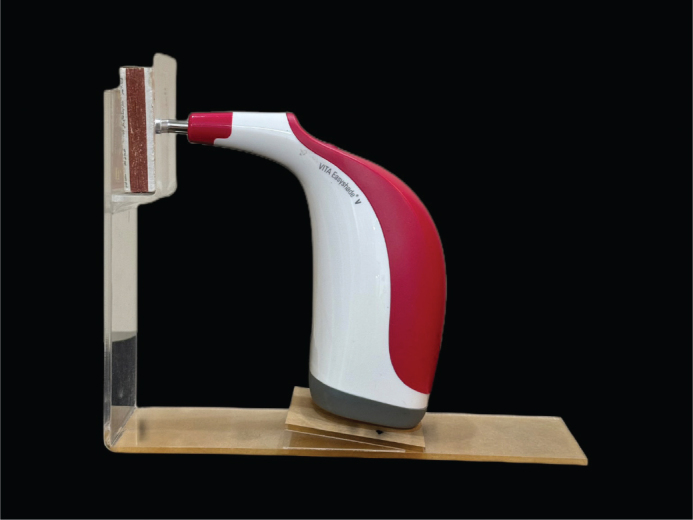
Device designed to ensure reproducible tooth positioning for color measurement.

The area used to determine the color of the teeth was at the interface of the cervical and middle thirds of the crowns in such a way that two-thirds of its height was in the cervical, and one-third was in the middle third of the tooth crown. Moreover, we designed equipment that held the spectrophotometer in a repeatable position and affixed the teeth in silicon blocks (Coltene, Altstatten, Switzerland) to ensure reproducible tooth positioning (Figure 3). In addition, the mean value of three times color measurements was considered at each step to reduce tool error.

Tooth color measurements occurred in two steps:

1. T1: After MTA-setting and complete restoration
2. T2: After thermocycling aging

Each sample’s color change (ΔE^*^) was calculated using the following equation [6].

ΔE = [(L_2_ – L_1_^*^)^2^ + (a_2_ – a_1_^*^)^2^ + (b_2_ – b_1_^*^)^2^]^0.5^

ΔL= L_2_ – L_1_

L* represents lightness (ranges in value from 0 (black) to 100 (white))

a* represents greenness/redness (negative values indicate green, positive values indicate red)

b* represents blueness/yellowness (negative values indicate blue, positive values indicate yellow).

### Statistical analysis

Data were assessed using SPSS software (PASW Statistics 18; SPSS Inc., Chicago, IL). Continuous variables with a normal distribution were presented as mean ± standard deviation. One-way ANOVA and two-way ANOVA were used to evaluate the effects of low and high-intensity LED light-curing on ΔL and ΔE. The level of statistical significance was set at 0.05 to assess interactions and the main effects.

## Results

**Figure 4:**
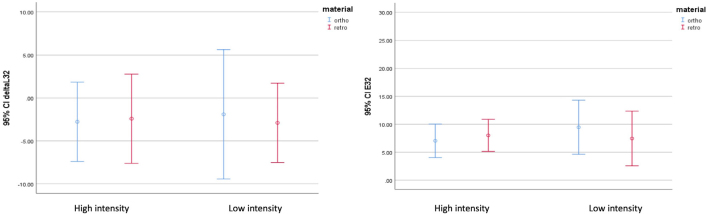
The result of a 95% confidence interval of ΔL and ΔE in Ortho MTA/Retro MTA groups in high/low intensities of light-cure (n = 40).

The mean and standard deviation of ∆E and ∆L values, representing the changes in both stages across all samples, are summarized in Table 2. The result of a 95% confidence interval of ΔL and ΔE in Ortho MTA/Retro MTA groups in high/low intensities of light-curing is presented in Figure 4. Moreover, none of the samples were lost.

**Table 2:** ΔL and ΔE values (mean ± SD) of each OrthoMTA/RetroMTA group in high/low intensity of light cure.

Subgroups	OH^†^	OL^‡^	RH^§^	RL^¶^
**∆L (Mean ± SD)**	−2.77 ± 6.88	−1.89 ± 10.53	−2.40 ± 7.27	−2.89 ± 6.46
**∆E (Mean ± SD)**	7.03 ± 4.46	9.47 ± 6.76	8.01 ± 4.01	7.45 ± 6.85

SD: standard deviation.

† OrthoMTA with high intensity of light-cure.

‡ OrthoMTA with low intensity of light-cure.

§ RetroMTA with high intensity of light-cure.

¶ RetroMTA with low intensity of light-cure.

∆L values were negative for all groups, indicating reduced lightness. No significant difference in ∆L values was found among the four groups (*p* = 0.992).

Coronal discoloration (∆E) was higher than 3.3 in all groups, demonstrating clinically perceivable changes [17]. No difference in ∆E was found among the four groups (*p* = 0.868).

The results of a two-way ANOVA of ∆E and ∆L values showed no significant effects of type of MTA or of light-cure intensity, and no interaction between the type of MTA and light-cure intensity (*p* > 0.05).

## Discussion

The present study evaluated coronal discoloration following the use of OrthoMTA and RetroMTA as intracanal barriers under two LED light-curing intensities. Although all groups showed clinically perceptible color change (ΔE > 3.3) and reduced lightness (ΔL) after thermocycling, no statistically significant difference was observed between the two intensities (1,200 mW/cm² vs. 3,000 mW/cm²). This suggests that when the total delivered energy is equal, curing intensity alone does not significantly influence discoloration induced by these HCSCs.

Bovine incisors were used to simulate the clinical situation by facilitating access to caries-free samples, as it is hard to access caries-free human teeth [18], and reducing variability related to human tooth morphology [19]. Bovine teeth are acceptable substitutes for human teeth in studies of crown discoloration and bonding to enamel and dentin [20]. However, their higher dentinal tubule density compared with human dentin [21] may have increased material penetration and, consequently, discoloration.

Samples were incubated for 7 days before coronal restoration to reduce the risk of MTA displacement [22]. MTA was covered with RMGI to minimize microleakage, and all teeth were restored with composite to better simulate clinical conditions.

According to the spectrophotometer manufacturer’s instructions, measurements must be performed with the tip at 90° to the surface and without movement. Although different fixation methods have been reported [6], we designed a custom device for standardized tooth positioning. This allowed all measurements to be obtained from the same location, improving reliability.

We designed the method to equalize the total energy obtained from the light-cure to minimize the effect of composite discoloration. Each sample received 48,000 J. In low-intensity subgroups, each tooth received 40 s of light with 1,200 mW/cm^2^; in high-intensity subgroups, each tooth received 16 s of light with 3,000 mW/cm^2^. The time set in low-intensity subgroups was due to the manufacturing structure. On the other hand, the duration of curing in the Eghbal et al. study was 160 or 40 s in human teeth with the Demetron Kerr light-cure unit. They did not state the intensity of the light-curing device in their study [23]. Similarly, Kang et al. irradiated HCSC discs for 15 or 30 min at 1,000 mW/cm² [2], durations far from clinical practice. They reported greater discoloration in materials containing bismuth oxide, whereas in the present study, no difference was observed between materials with and without bismuth oxide.

Other studies have shown that prolonged light exposure and anaerobic conditions can affect discoloration of MTA-based materials [24]. Comparison of different curing units also demonstrated variable discoloration outcomes, although evaluations were performed after short periods (5 days) and under oxygen-free conditions, which may have influenced results [8]. Differences in curing duration and environmental conditions likely explain discrepancies among studies.

Various methods have been used for color assessment, including spectrophotometry [25], colorimetry [26], visual assessment [27], and digital image analysis [23]. Spectrophotometry, combined with the CIE Lab* system, offers high sensitivity and standardization [27] and was therefore selected for this study.

In the CIE Lab* system, overall color change (ΔE) reflects combined variations in L*, a*, and b* and is more representative than individual parameters [28, 29]. Both OrthoMTA and RetroMTA groups showed increased ΔE after thermocycling. Discoloration may be attributed to material interaction with dentin or penetration into dentinal tubules. However, the use of RMGI and composite restoration may have limited the extent of discoloration. Differences from studies reporting severe discoloration after light irradiation [24] may be related to the absence of blood, the use of teeth instead of discs, and the presence of RMGI. However, using distilled water with propylene glycol has been found to reduce the effect of the blood on the color alteration, and the use of distilled water should therefore be considered for future studies [30].

All samples demonstrated decreased ΔL values over time, indicating a reduction of luminosity and a trend toward darkening. Similar reductions in ΔL with increased light exposure were reported by Eghbal et al. [23] and Valles et al. [8]. It should be noted that the current study duration was a simulation equivalent of 6 clinical months. But the studies of Eghbal and Valles et al. lasted 28 and 5 days, respectively.

Comparison between materials showed no significant difference in ΔL or ΔE between OrthoMTA and RetroMTA. This agrees with studies reporting no significant difference in discoloration among various calcium silicate–based materials used as intracanal barriers [31] and even in the presence of blood. However, in the absence of blood, some materials, such as Biodentine and EndoSequence, have shown less discoloration than OrthoMTA [6].

This ex vivo model aimed to simulate clinical VPT conditions while evaluating the effect of curing intensity on MTA-induced discoloration. Although perceptible discoloration occurred, differences between groups were not significant. Several factors may explain this finding: the relatively short study duration, as delayed discoloration has been reported at 1 year [32]; sample thickness, which was not standardized to a uniform labial wall thickness as in some studies [33]; the use of RMGI, which may have reduced contact between discoloring agents and dentin [34]; inherent differences between ex vivo and intraoral conditions; limitations in achieving a perfectly flat labial surface for spectrophotometric assessment; and the use of equal total light energy despite manufacturer recommendations of shorter curing times. Moreover, using samples free of MTA as negative controls would have strengthened the design. Future studies should consider varying curing durations and longer evaluation periods.

## Conclusions

Based on the results of the present study, we conclude that curing with high- or low-intensity with the same amount of energy does not significantly change the color of teeth treated with RetroMTA or OrthoMTA cement. However, all groups presented ∆E above the clinically perceptible value (∆E ≥ 3.3) and decreased ∆L values.

## Data Availability

The data that support the findings of this study are available on request from the corresponding author. The data are not publicly available due to privacy or ethical restrictions.
